# Digital psychoeducational rehabilitation supported by serious games for psychiatric inpatients: a protocol study

**DOI:** 10.3389/fpsyg.2026.1881403

**Published:** 2026-09-18

**Authors:** Antonella Gigantesco, Umberto Volpe, Gemma Calamandrei, Laura Orsolini, Alessandra Premici, Vindice Deplano, Debora Del Re, Isabella Cascavilla, Sonia Brescianini, Laura Camoni

**Affiliations:** 1 Centre for Behavioural Sciences and Mental Health, National Institute of Health, Rome, Italy; 2 Unit of Clinical Psychiatry, Polytechnic University of Marche, Ancona, Italy

**Keywords:** digital mental health, integrated interventions, psychoeducation, schizophrenia spectrum disorder (SSD), serious games, youth mental health

## Abstract

**Introduction:**

Schizophrenia spectrum disorders (SSD) are characterized by impairments in thought, perception, emotion, and behavior, leading to reduced personal and social functioning. While pharmacological treatments have limited efficacy on negative and cognitive symptoms, psychosocial interventions play a crucial role but remain difficult for many patients to access, particularly among youths. Digital interventions, particularly serious games, offer a promising approach to enhance recovery by improving accessibility, engagement, and motivation. The present protocol study aims to develop and preliminary evaluate serious games as a complementary tool to standard psychoeducational interventions for young SSD patients. The primary objective is to evaluate the feasibility of some serious games in a real-world clinical setting. Secondary objectives are to explore whether the addition of those serious games to a standard psychoeducational intervention leads to a greater improvement in global functioning, emotion dysregulation, severity of general psychopathology and other clinical or functional outcomes.

**Methods:**

A quasi-experimental, non-randomized study with a historical control group will be conducted at the Unit of Clinical Psychiatry, Polytechnic University of Marche (Italy), involving patients aged 15–24 years diagnosed with SSD and related disorders, including at-risk mental states. Participants will be inpatients who had received a standard conventional psychoeducational program (control group) and inpatients who will receive the same program supplemented with serious games (experimental group). These games delivered both via web-based platform and smartphone application, simulate real-life scenarios and reinforce learning through interactive and repetitive exercises.

**Results:**

We expect the integration of serious games to be feasible and well accepted, enhancing engagement, adherence, mental health literacy, and the consolidation and transfer of skills acquired through standard psychoeducation, with potential improvements in global functioning and psychopathological symptoms.

**Conclusion:**

This approach may represent a scalable and innovative strategy to improve the recovery and long-term outcomes in youth with SSD and related disorders admitted to an acute psychiatric setting.

## Introduction

Schizophrenia and schizophrenia-related disorders (i.e., schizoaffective disorder, schizophreniform disorder, and other psychotic disorders) are severe mental illnesses (SMIs) affecting more than 24 million people worldwide, with profound consequences on cognition, perception, affect, behavior, and overall functioning (WHO Schizophrenia, 2022). Their chronic course is often associated with worsening cognitive and negative symptoms, which are major determinants of functional disability, reduced autonomy, and impaired social, emotional, and daily functioning (Cheng and Yen, 2021). Social cognition deficits further compromise interpersonal relationships, vocational outcomes, and community adjustment (Orsolini et al., 2022; Carling et al., 2025). Since pharmacological treatments have limited efficacy on cognitive and negative symptoms (Damiani et al., 2026), multidisciplinary rehabilitation combining psychotherapy with psychosocial interventions—including psychoeducation, cognitive training, and social and life skills training—is recommended to improve functional recovery and independent living (Rocca et al., 2021). However, access to these interventions remains uneven due to differences in healthcare resources, workforce availability, and geographical factors.

Early psychosocial interventions are particularly important for young people with schizophrenia spectrum disorders (SSD) and those at clinical high risk for psychosis (CHR-P) (Camacho et al., 2026). The CHR-P paradigm identifies individuals with attenuated psychotic symptoms, brief intermittent psychotic episodes, genetic vulnerability, or basic symptoms who are at increased risk of developing psychosis (Salazar de Pablo et al., 2021; Camacho et al., 2026). Approximately 25% of CHR-P individuals move to psychosis within 2–3 years, with the risk increasing over time, while nearly one-third of non-converters continue to experience persistent functional impairment (Salazar de Pablo et al., 2021; Takahashi et al., 2017).

Therefore, implementing and integrating in clinical practice interventions specifically targeting social cognition and functional impairment is crucial to improve clinical and functional outcomes, as well as greatly reduce the transition toward severe impairment and psychopathology, particularly in youth mental health (Orsolini et al., 2021, 2022). Psychosocial interventions, including psychoeducational intervention, are performed by repetition and simulation, in sessions supervised by rehabilitation technicians or medical staff. This implies that the patient moves frequently to the rehabilitation facilities and adjusts the schedule accordingly. Rehabilitation supported by digital technologies emerges as a possible solution to improve and facilitate access to care and assist psychiatric patients in the rehabilitation process (Orsolini et al., 2025). It also emerges as a solution to provide high-intensity, repetitive, task-specific exercises which can be used, as booster sessions, on their own at home by inpatients, to enhance the rehabilitation process initiated during a hospitalization. Consistently, using educational technology (i.e., virtual reality, video games, and other applications) in conjunction with traditional evidence-based interventions demonstrates promising results to improve personal and social skills functioning of psychiatric patients (Surdyka et al., 2023). Because of the significant value of intrinsic motivation which may exert in the rehabilitation process, it is also important to evaluate tools and approaches that foster patient's motivation and involvement, including retention rate (Altman et al., 2023; Hu et al., 2025). Gamification is known for its potential in increasing motivation and providing rich learning experiences. Gaming software could elicit emotional and motivational stimuli and foster the development of interpersonal skills in real-world situations (Choi et al., 2020).

Serious games—games designed for educational purposes—are usually used to indicate that their positive effects on motivation are not the main reasons for using games, aiming at training and learning, instead (Del Vecchio et al., 2024). The available evidence suggests that serious games could be an effective format for an intervention to reduce psychiatric symptoms and improve several functional outcomes of patients (Dewhirst et al., 2022). For example, serious games interventions have already been used in a range of settings aiming to improve symptoms and functional outcomes in schizophrenia (Choi et al., 2020; Amado et al., 2016). Such studies demonstrate the potential utility of serious games as both a stand-alone and supplementary treatment tool within the context of various psychosocial interventions.

Given the potentialities of the implementation of digital mental health in the context of psychiatric rehabilitation, particularly in youth mental health and in early stages of SSD as well as among CHR-P population, the current project aims at addressing the use of serious games in the context of psychoeducational rehabilitation techniques, to contribute to the promotion of well-being and health in a holistic perspective, focusing mainly on personal and social functioning in youths diagnosed with SSD and related disorders, including CHR-P. This research protocol study proposes the use of serious games in psychoeducational interventions delivered in public inpatient psychiatric services, with the aim of promoting well-being and strengthening awareness of the disorder, fostering a greater sense of control over one's experiences and overall improving patients' global functioning.

## Methods

### Study design

Primarily, a feasibility study will be conducted. Secondarily, a preliminary effectiveness study will be conducted for investigating the effects of serious games, as a supplement to conventional psychoeducational interventions, on some clinical and functional outcomes in a cohort of adolescents and young adults (aged 15–24) inpatients, to obtain preliminary estimates and inform the design and sample size calculation of a future definitive trial.

This preliminary effectiveness study will adopt a quasi-experimental, non-randomized design with a historical control group. The experimental group will comprise at least 30 consecutive patients who will receive the standard rehabilitation program supplemented with serious games. The control group will consist of 30 consecutive patients who had undergone the standard rehabilitation program over the three-month period preceding the implementation of serious games. The two groups will be compared at baseline in terms of socio-demographic variables and clinical severity. Variables for which the two groups will differ will be included in the models. With a total sample size of *N* = 60 (*n* = 30 per group), the study is powered (1-beta = 0.80, alpha = 0.05) to detect an exact medium-to-large interaction effect size of Cohen's *d* = 0.7356 (equivalent to Cohen's *f* = 0.3678), ensuring adequate sensitivity to capture clinically meaningful differences in functional recovery trajectories. With this sample size we will be able to detect an improvement in the Global Assessment of Functioning (GAF) scale score in the treated sample of about 7 points higher. We computed the exact effect size using G*Power (Version 3.1) (Faul et al., 2007).

It should be noted that throughout the study period, the rehabilitation setting, the standard treatment protocol, healthcare professionals, and clinical pathways were planned to remain unchanged. The only planned modification to the rehabilitation program is the addition of the serious games intervention for patients enrolled after its implementation.

### Outcomes

#### Primary outcomes

Primary outcomes include participant retention, adherence to the serious game's intervention, and intervention completion.

#### Secondary outcomes

Secondary outcomes include global functioning, anxiety and depression symptomatology, emotion regulation, severity of psychopathology, use of coping strategies, internalized stigma, mental health knowledge, cognitive functioning and problem-solving skills.

### Setting

#### Inclusion criteria

Participants will include patients admitted consecutively to the Acute Unit of Clinical Psychiatry, Department of Neurosciences (DIMSC), Polytechnic University of Marche, Ancona, Italy.

Inclusion criteria comprise: (a) aged 15–24 years-old; (b) having a diagnosis of SSD and/or related conditions, such as schizophrenia, schizotypal disorder, delusional disorders, schizoaffective disorders, other psychoses, including CHR-P, according to the criteria of the International Statistical Classification of Diseases and Related Health Problems-10th revision (World Health Organization, 2004); (c) participation in the conventional psychoeducational program during the hospitalization at the Unit of Transition Psychiatry, University Hospital of Marche Region, Ancona; (d) Internet access; (e) ability to fluently speak, read and understand Italian language; (f) having provided a signed written informed consent to participate in the study (only for the experimental group).

#### Exclusion criteria

Patients cannot be included in the project if they have: (a) an intellectual disability, difficulty understanding Italian, learning difficulties, or moderate-to-severe cognitive deficits; (b) current uncontrollable psychomotor agitation, or current drug and/or alcohol intoxication that could interfere with attention and/or concentration during psychoeducational sessions; or, (c) acute psychiatric symptoms that may affect consciousness or cause temporary changes in cognitive ability.

### Procedures

Participant recruitment for the experimental group began in June 2026 and is anticipated to last approximately 3 months, with the intervention commencing immediately after enrolment and baseline assessment. As anticipated, the control group will consist of consecutive patients who had undergone the conventional psychoeducational program from March to May 2026 (without the serious games' addiction).

For all patients in the experimental and control groups, sociodemographic data (gender, year of birth, nationality, educational attainment, marital status, employment status) and basic clinical data (diagnosis, age at onset, duration of illness, number of previous admissions, duration of the most recent admission, medication regimen) are collected upon admission, for each patient using a specially designed case report form (CRF).

Personal data will be stored and processed in compliance with applicable data protection regulations on a secure server of the Ancona Hospital. Before being transferred to the Italian National Institute of Health (Istituto Superiore di Sanità, ISS) for statistical analyses, all data will be pseudonymized.

Specifically, each participant will be assigned a unique study identification code that replaces all directly identifying personal information in the research dataset. The key linking the study identification code to participants' identities is stored separately on a secure server at the Ancona Hospital, is protected by appropriate technical and organizational security measures, and is accessible only to the Principal Investigator and authorized study personnel.

Access to identifiable data will be restricted to the study investigators and authorized study personnel only.

Serious game usage data (e.g., login frequency and game completion status) will be linked to participants' clinical data using the same pseudonymized study identification code, ensuring that no directly identifiable personal information is included in the research dataset.

The study complies with the General Data Protection Regulation (GDPR), and appropriate technical and organizational measures have been implemented to ensure data security, including secure data storage, controlled access, password-protected user credentials, and cybersecurity safeguards. A Data Protection Impact Assessment (DPIA) was conducted as part of the study preparation. No independent cybersecurity audit or penetration testing was performed as part of the study.

Before submission to the National Ethics Committee, the study protocol was reviewed and approved by the Data Protection Officer (DPO) of the ISS, confirming compliance with applicable data protection requirements.

#### Conventional psychoeducational program

A routine group psychoeducational program based on the Falloon approach (Falloon et al., 1984) is offered to acute inpatients admitted to the Transition Psychiatry Ward, at the Unit of Clinical Psychiatry, Department of Neurosciences/DIMSC, University Hospital of Marche Region, Polytechnic University of Marche, Ancona, Italy. This is a cognitive-behavioral approach within the stress-vulnerability model and coping-oriented program (Fowler et al., 1995). Specifically, it combines the disorder management strategies of Falloon's psychoeducational approach (Falloon et al., 1984) with the model of individual cognitive restructuring therapy for psychosis (Fowler et al., 1995). In Falloon's approach, in particular, psychiatric patients are trained to use structured problem-solving strategies and other strategies to tackle the problems caused by the most stressful situations in their lives, and to use their social network to obtain support from those people most willing and able to help them to solve problems. Group sessions are provided to teach inpatients to recognize stressful events situations and to identify their early warning signs. Key strategies and skills include emotional recognition, emotional expression, and effective communication skills and structured problem solving. Persistent symptoms are normalized as experiences that most people may have when subjected to extreme biological or psychosocial stress. Personal strengths of inpatients are reinforced, rather than their weaknesses and disabilities. The psychoeducational intervention has been already adapted by the research team of the Polytechnic University of Marche to be delivered to youth (aged 15–24) patients consecutively admitted to the Transition Psychiatry unit.

The psychoeducational program is delivered by trained staff of the Clinical Psychiatry Unit (psychiatrists, psychologists, or rehabilitation therapists), according to a standardized manual which includes the topics and modules of the program sessions. The same program but supplemented with the serious games is scheduled to be delivered by trained staff from the Unit, with analogous specializations. The topics of the program sessions are described in Table 1 (in Supplemental materials).

#### Serious games' program (conventional plus serious games)

It includes the incorporation of serious games, which are added to the conventional program with the aim of reinforcing the learning of information received by patients during face-to-face sessions with the clinicians leading the psychoeducational program. These games will essentially concern the exercises assigned at the end of each session, which are normally carried out by completing paper forms handed out to patients at the end of each session. Serious games will offer an alternative to paper forms to facilitate the completion of the so-called homework, which is very often ignored or completed with difficulty by patients.

#### General properties of the serious games

Up to June 2026, three serious games had been fully developed, addressing the domains of basic emotions, hallucinations, and problem-solving, respectively.

The games were conceptually designed by researchers from the Italian National Institute of Health (Istituto Superiore di Sanità, ISS) with expertise in psychiatry, clinical psychology and psychoeducational interventions (Authors: AG, LC, GC). The ISS researchers developed the educational contents, game narratives, scenarios, dialogues, and gameplay requirements, ensuring consistency with the underlying clinical framework.

The software programming and development have been carried out by the software engineer VD (co-author), who acted as the technical developer in close collaboration with the ISS research team, which include the above-mentioned researchers and two research technicians (Authors: DD and IC) and a research clinician of the Ancona Unit staff (Author: AP).

The development process was iterative, with regular meetings between the technical developer and the ISS research team to refine game mechanics, usability, and functionality according to the predefined clinical and educational objectives. The final version of the games was reviewed and approved by the whole project team (ISS plus Ancona unit) before being used in the study.

The intellectual property of the serious games, including the clinical contents, narratives, and game design, belongs to the ISS researchers who conceived the intervention, while the software source code was implemented by the technical developer within the framework of the project funded through a research grant.

The software developer received financial compensation for his technical work through the project funds. No commercial company was involved in the development, commercialization, or ownership of the software.

The games include short stories, with interactive animated scenes in which the actors experience situations that may cause problems to resolve in daily activities. As the story unfolds, and after each scene, the user is queried about what happened, and which strategies he would use in the place of actors. Games can take different paths depending on the choices the player makes.

The games provide user feedback and, in the event of errors, display a hyperlink with information, education and strategies, whose objective is to help users understand the scene that they have just watched and apply effective coping strategies.

The progression of the scenario depends directly on the user's choices and may vary considerably across players. Unlike conventional video content, there is no fixed progression timeline; instead, the game state is continuously saved, allowing users to pause the session and resume it later from the exact point at which they stopped.

Participants can access the games via their own personal computer, tablet, or smartphone by logging into the platform using a unique username and password. Interaction with the game occurs through multiple modalities, including clicking on interactive objects, navigating between virtual environments, answering questions, completing data-entry forms, and engaging in dialogues with virtual characters. These characters are represented by static images with limited facial animations (e.g., mouth and eye movements) and communicate through professionally pre-recorded audio. At predefined points in the narrative, users are prompted to select one of several response options, with subsequent events determined by their choices.

#### Contents of the serious games

The games feature four teenagers—two girls and two boys, aged 16–18—and a doctor who steps in at the end of the game to summarize the choices made by the players and discuss them critically. Each serious game lasts approximately 20–30 min.

- The first game (“Going to the cinema”) Figure 1 focuses on managing emotions (specifically anger and anxiety). The goal of the game is to teach players to recognize emotions, connect emotions to corresponding thoughts, and interpret emotional experiences using Ellis's ABC model (activating event-belief-consequences) (Ellis, 1957).

**Figure 1:**
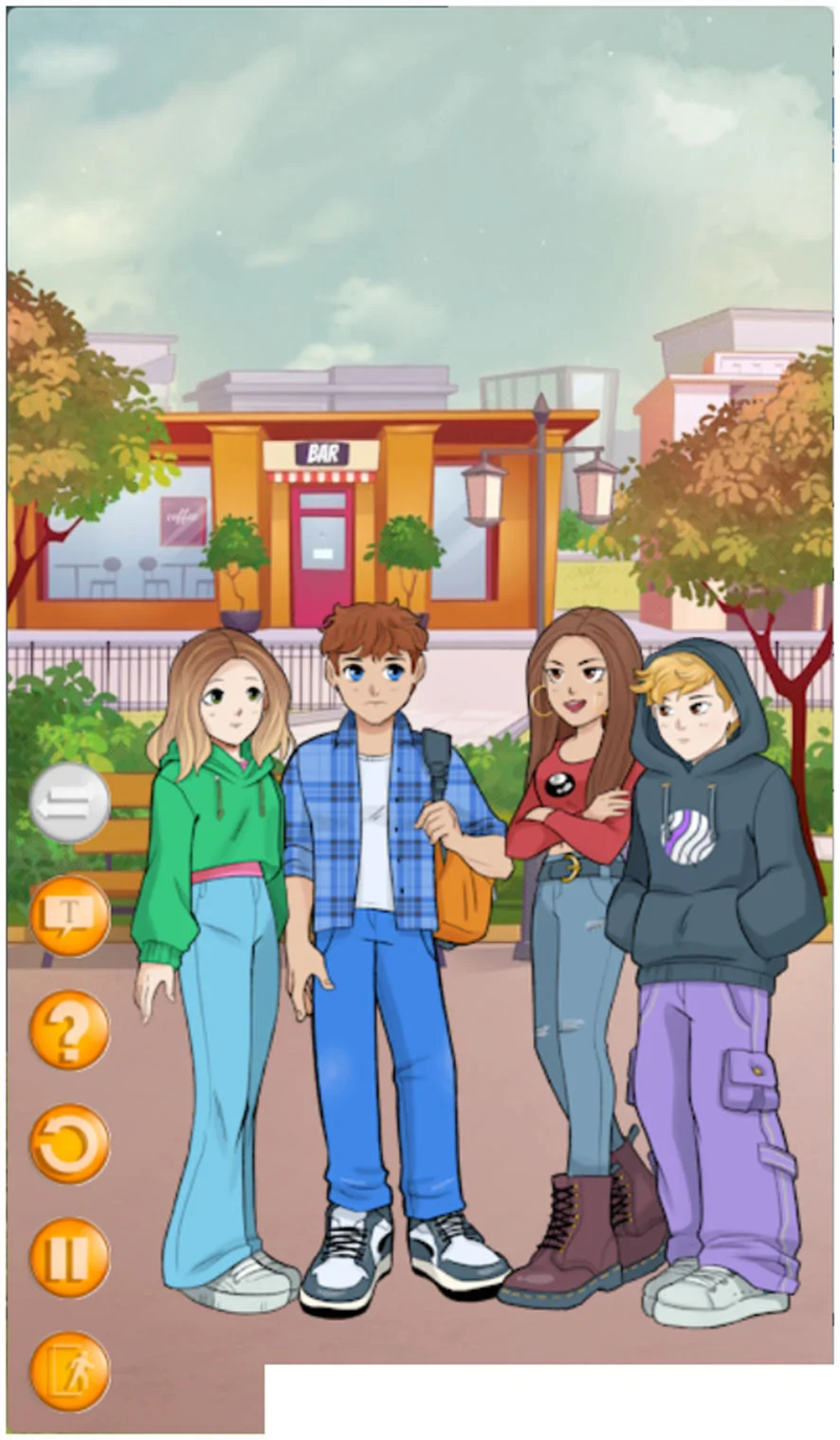
Going to the cinema.

*Storyline*

The four friends decide to spend an evening together by going to the cinema. They agreed to meet in front of the cinema entrance, but at the appointed time, only two of them show up. The minutes tick by, and there's no sign of the other friends. The wait drags on, and the silence gives way to questions, speculation, and worry. One of the two friends grows up increasingly anxious, fuelled by the fear that something serious might have happened; the other, however, is overcome by anger, interpreting the delay as a lack of respect for their agreed-upon plans. When the two latecomers finally arrive, they do so calmly and casually, explaining that a simple unexpected event held them up longer than expected, unaware of the emotions and assumptions their absence had stirred in their friends.

Doctor concludes the game by pointing out that the way you assess events, manage negative feelings internally, and then express them (or choose not to) influences others' behavior and how you feel afterward.

- The second game (“Out of Control”) Figure 2 focuses on hallucinations. The goal of the game is to help participants understand what ‘hallucinations' are, providing them with the tools to recognize and deal with them in a mindful and effective way.

**Figure 2:**
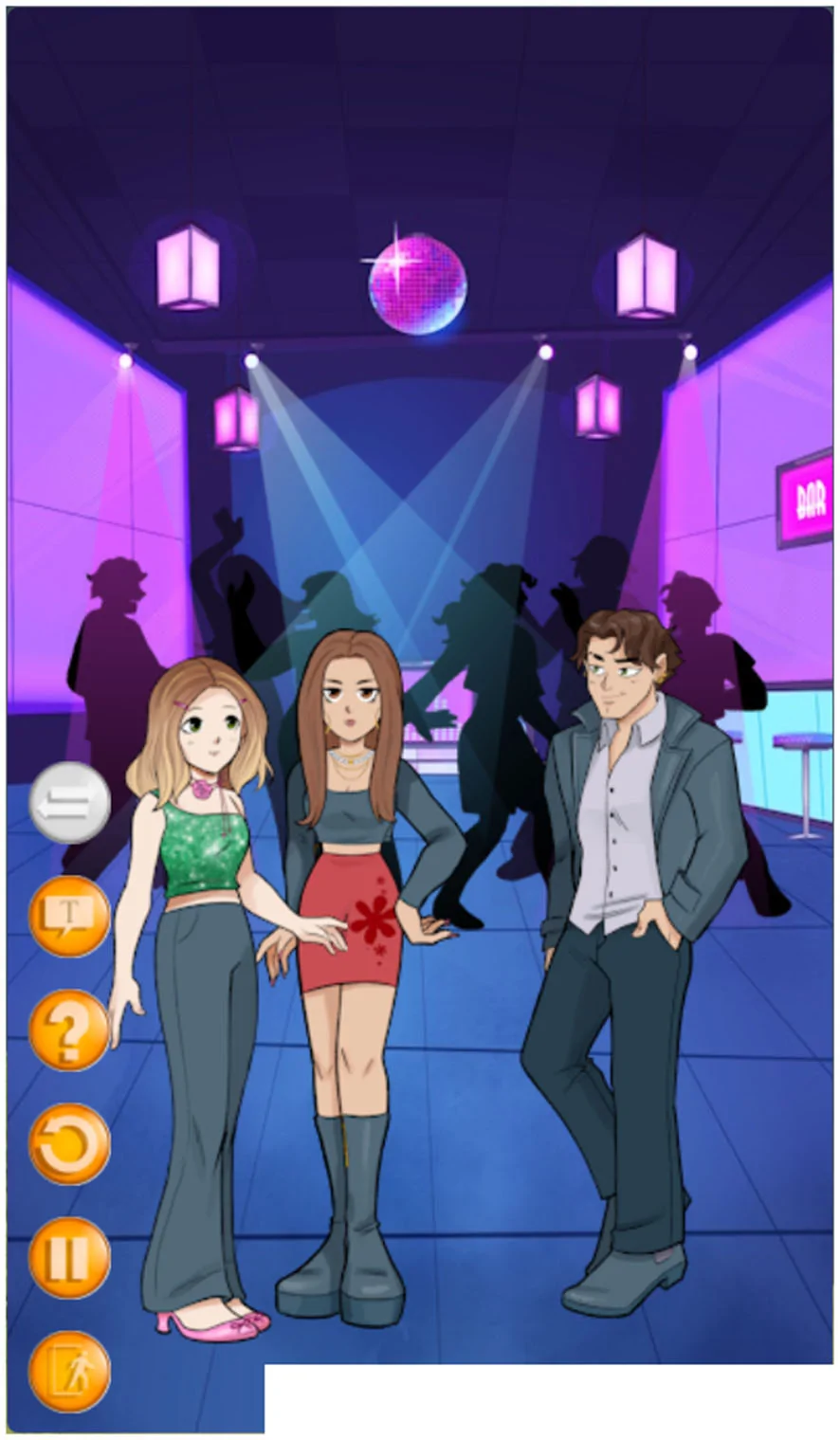
Out of control.

*Storyline*

The teens decide to go to a nightclub on a Saturday night to celebrate the end of the school year. At the club, they dance and have fun. After a few hours, some of them are tired and want to go home, but one girl wants to stay. They meet a guy who offers them something to “get them going”—some supplements that won't do any harm. The girl decides to try it, and after a while she begins to hallucinate; eventually, the euphoria gives way to mistrust and fear.

Doctor concludes the game by sharing some educational messages about perception, psychotic symptoms, and substance use, as well as self-regulation techniques that can be helpful when “high.”

- In the third game (“Friends on the ball”), Figure 3 the players put their problem-solving skills to the test. The goal of the game is to teach the main steps of problem-solving: defining the problem, generating possible solutions, evaluating their advantages and disadvantages, choosing the most appropriate alternative, planning the action, and verifying the results. Through this process, players learn to tackle problems and achieve goals effectively and rationally by critically evaluating the various available options and choosing the most appropriate solution, rather than acting impulsively.

**Figure 3:**
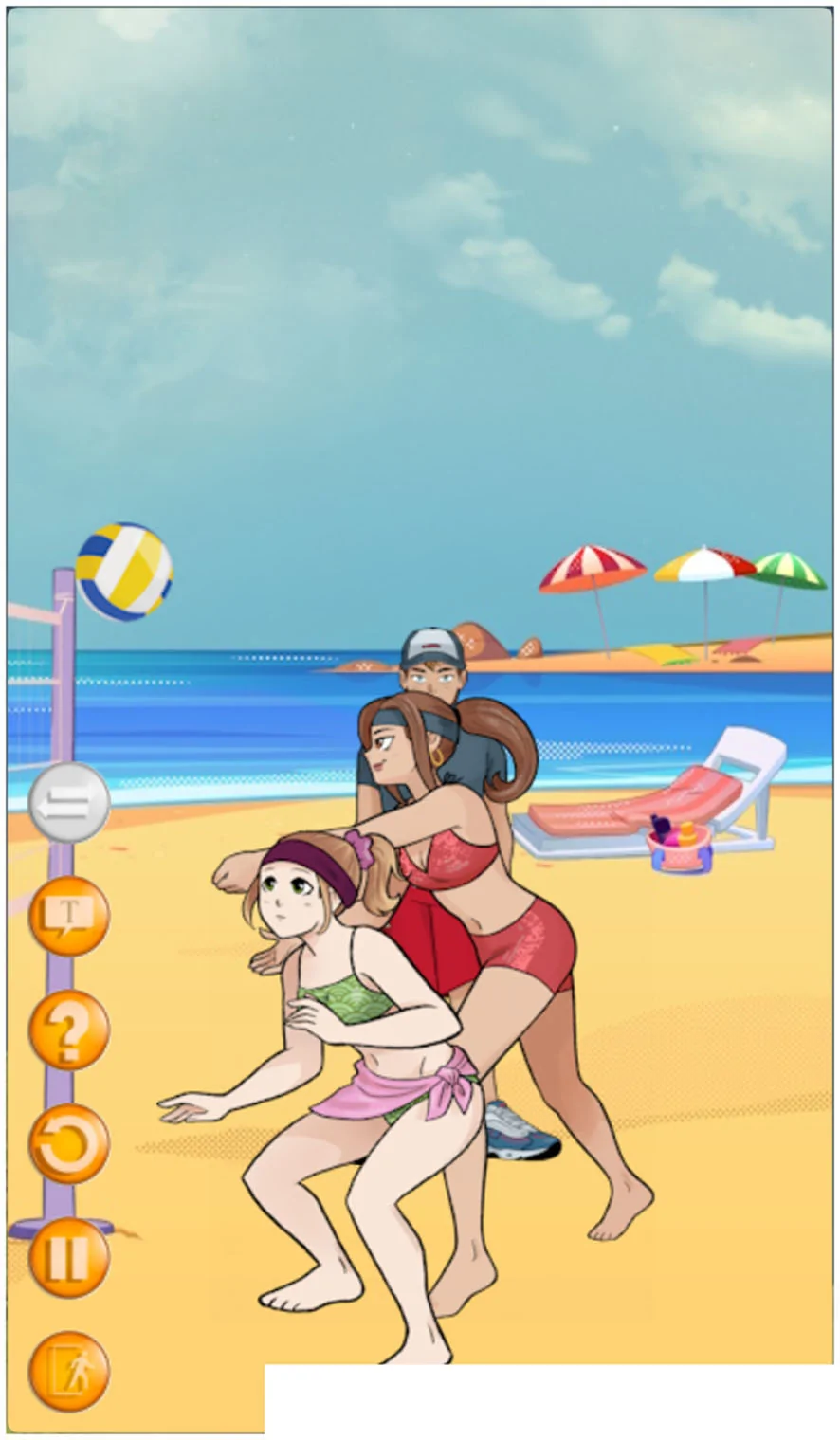
Friends on the ball.

*Storyline*

The four friends are playing beach volleyball and are invited to participate in a tournament featuring teams from all over the city. Not everyone plays well, so they begin to debate whether those who are less skilled should also participate in the tournament. Two of them are torn: they'd like to participate, they discuss it, but in the end, they make different choices.

Doctor concludes the game by analyzing each player's choices and determining which would be the best one.

Other remaining serious games, which taken together, will cover all the topics addressed by the conventional psychoeducational intervention, are still in the prototype development phase.

#### Technical characteristics of serious games

The serious games were developed using standard web technologies (HTML, JavaScript, and CSS) compatible with all major web browsers, without requiring additional plugins. To facilitate accessibility across different devices, each game was developed in three versions:

- A native SCORM-compliant learning object, designed for delivery through a Learning Management System (e.g., Moodle) to enable learner tracking, or alternatively deployable on a standard web server.
- An Android application for smartphones.
- A standalone executable version for Windows-based personal computers that does not require installation.

Across all versions, the user interface adopts a vertical (portrait) layout optimized for smartphone use.

ISS technical computer system staff assists managing technical problems, such as login failures, software bugs, device incompatibility, unstable Internet connection, or interruption of game sessions. No formal usability study was conducted before implementation. However, during the development phase, the research team interacted with the games and checked their functionality. No formal pilot study with a separate sample of patients was conducted before the implementation of the intervention. No formal software validation according to specific software-engineering or medical-device standards was performed. The games were nevertheless tested during development to verify that the main functions worked as intended and that the games could be used as planned in the intervention. Technical functioning was checked during development. In particular, the research team verified that the games operated correctly and that the intended activities could be completed. No study-specific cybersecurity incident-management procedure was established. However, any such incidents involving the institutional infrastructure would fall under the standard IT security and incident-management procedures of the ISS.

#### How serious games will be implemented as part of the psychoeducational program

The sessions are conducted three times per week throughout the participants' hospital stay and are delivered individually to each participant by a facilitator (psychiatrist, psychologist, rehabilitation therapist, or professional educator) supported by a co-facilitator (psychiatry resident or professional educator).

During each session, the facilitator introduces the serious game assigned for that week, explaining its psychoeducational objectives and providing participants with instructions on how to access and use it. Participants are then asked to complete the game independently as homework before the following session. Patients have to complete the game at least once, but they may also play it more times if they like. All participants receive the same serious games, with identical core content, structure, educational objectives, and predefined duration. Within each game, participants can make different choices, resulting in different feedback or sequences; however, these adaptive pathways do not modify the intervention dose or core psychoeducational content.

Each session, including the presentation of the serious game, lasts approximately 90 min. Participants' engagement with the serious games is monitored through the digital platform, which records usage data, including the number of logins and the completion status of each game. Homework completion was monitored through the intervention platform, which recorded login frequency and completion status; incomplete activities were addressed during the subsequent session as appropriate.

The collected usage data consist of the number of accesses recorded for each game. These data will be used to quantify participants' interaction with the different games and to analyse usage patterns.

Therefore, patients in the experimental group are informed about the availability of the serious games and their entirely voluntary use. Participants do not receive more time on treatment-related activities provided by clinicians than those in the control group, and clinicians do not provide additional attention or guidance beyond that received by the control group. As previously mentioned, each of the three serious games was designed to have an expected duration of approximately 20–30 minutes. Because the games are accessed through an online platform, participants' use of the games can be monitored objectively. For each game, the platform allows the researchers to determine whether the participant has accessed and completed the game. These data provide an objective measure of participants' exposure to the intervention. No additional minimum exposure criterion was established a priori. Therefore, adherence is assessed descriptively based on the number of games accessed and completed.

### Clinical outcome measures and timing

The following measures of psychopathology and functioning will be obtained for all patients included in the study, before the start of the conventional psychoeducation intervention and at the end of the entire intervention cycle:

1. Sociodemographic data (gender, year of birth, nationality, education, marital status, employment status) and baseline clinical data (diagnosis, age of onset, duration of illness, number of previous hospitalizations, length of last hospitalization, medication regimen).
2. Anxiety and depression symptomatology using the HADS which is a 14-item self-report measure able to assess the anxiety and depression levels of participants (Zigmond and Snaith, 1983; Costantini et al., 1999). The HADS is constituted by seven items relating to anxiety (anxiety subscale) and seven items relating to depression (depression subscale). Items are answered in a four-point response format with a total score ranging from 0 to 21 for each of the two subscales: the higher the score, the more severe anxiety or depression.
3. Emotional dysregulation using the Difficulties in Emotion Regulation Scale (DERS) (Gratz and Roemer, 2004; Giromini et al., 2012). This is a 36-item self-report questionnaire that assesses emotional dysregulation and difficulty regulating negative emotions through six subscales: non-acceptance, goals, impulsivity, awareness, strategies, and clarity. It measures how individuals respond to distress, with higher scores (36–180) indicating greater difficulty regulating emotions.
4. Severity of general psychopathology; used transversally in psychosis, affective disorders and in all contexts of clinical and research psychiatry to monitor symptomatic change, assessed with the Brief Psychiatric Rating Scale (BPRS) (Overall and Gorham, 1962; Ventura et al., 2000; Morosini and Casacchia, 1995; Roncone et al., 1999). It consists of 24 items that explore both symptoms and signs of psychopathology. The scale provides a rating from 1 (No symptom) to 7 (Extremely severe) for each item.
5. Overall severity of psychiatric illness, with the scale Clinical Global Impression (CGI-S) (Guy, 1976; Busner and Targum, 2007; Morosini and Casacchia, 1995). The CGI considers the patient's medical history, psychosocial circumstances, symptoms, behavior, and the impact of symptoms on the patient's functioning. The assessment consists of a 7-point scale (1 = Normal; 7 = Among the very ill patients).
6. Global improvement or worsening of psychiatric condition since the start of treatment, with the Clinical Global Impression (CGI-I) (Guy, 1976; Morosini and Casacchia, 1995) a 7-point scale to assess the improvement or worsening of a patient's symptoms compared to baseline (start of treatment). It measures global change, with scores ranging from 1 (much improved) to 7 (much worsened).
7. Dispositional coping strategies through the Coping Orientation to Problems Experienced (Carver et al., 1989)—New Italian Version (COPE-NVI) developed by Sica et al. (2008). It is a self-assessment questionnaire composed of 60 items that identifies five solid and independent coping dimensions: social support, avoidance strategies, positive attitude, problem solving and recourse to religion.
8. Global psychological, social, and occupational functioning. A scale included in Axis V of the DSM-IV (American Psychiatric Association, 1994) provides a summary index of the level of functional impairment, explored through the Global Assessment of Functioning (GAF) scale (Endicott et al., 1976), which provides a score ranging from 0 to 100, with higher scores being associated with higher functioning.
9. Internalized stigma regarding one's mental illness with the Internalized Stigma of Mental Illness Inventory (ISMI) (Ritsher et al., 2003; Boyd et al., 2014), a self-assessment instrument with 10 items on a 1–4 Likert scale. The total score ranges from 1 to 4 (item mean), where higher scores indicate more severe stigma. It assesses five dimensions (alienation, stereotype endorsement, perceived discrimination, social withdrawal, stigma resistance).
10. Assessment of stigma-related mental health knowledge with the Mental Health Knowledge Schedule (MAKS) (Evans-Lacko et al., 2010; Pingani et al., 2019), composed of 12 items on a 5-point Likert scale; it focuses on six areas: help seeking, recognition, support, employment, treatment, and recovery. A higher total score indicates greater knowledge of mental illnesses and a lower tendency to stigmatize.
11. Cognitive functions through the Montreal Cognitive Assessment (MoCA) (Nasreddine et al., 2005; Conti et al., 2015; Santangelo et al., 2015) which explores mild cognitive impairment through the analysis of the domains visuospatial/executive, naming; memory; attention/concentration/working memory; language; abstraction; delayed recall; orientation, for a maximum total score of 30 points.
12. Problem-solving skills and goal achievement in a group context (tool internally adapted for psychoeducational intervention). The phased structure (problem definition, solution generation, pro/con evaluation, choice, implementation, verification) reflects the classic structured problem-solving model of Falloon (1988), Falloon et al. (1984, 1998) and Morosini and Casacchia (1995). Specifically, the assessment schedule timing was conceived as follows (Morosini et al., 1991): Baseline: after enrolment and before starting the intervention; Post-intervention: at discharge, or, when the assessment cannot be completed before discharge, at the first scheduled post-discharge visit, generally approximately 15 days after discharge; Long-term follow-up: not planned. Unlike protocols designed to evaluate longer-term clinical efficacy, which includes assessments at 3, 6 and 12 months, our study was primarily designed as a feasibility/preliminary study conducted during the acute inpatient treatment period. Therefore, no longer-term follow-up is planned.

#### Statistical analysis

Data on sociodemographic and clinical characteristics will be summarized using descriptive statistics. Continuous variables will be presented as means and standard deviations or medians and interquartile ranges, as appropriate, whereas categorical variables will be reported as frequencies and percentages. Baseline comparability between the prospective intervention group and the historical control group will be assessed using independent-samples *t*-tests or Mann–Whitney *U*-tests for continuous variables, and chi-2 or Fisher's exact tests for categorical variables, as appropriate.

The primary objectives of this study are to evaluate the feasibility and acceptability of integrating *ad hoc* developed serious games into standard psychoeducation in an inpatient clinical setting. Primary outcomes include participant retention, defined as the percentage of enrolled participants completing the post-intervention assessment, adherence, quantified as the number of prescribed serious game sessions completed by each participant, and intervention completion, defined as the proportion of participants completing all prescribed game sessions. A 75% retention rate and an 80% completion rate were chosen, as they are common benchmark targets in clinical research. The number of consecutive eligible patients approached, the number who agree to participate, and the number who decline will be also reported. As this was a feasibility study, no formal anticipated refusal rate was prespecified. Rather, the proportion of eligible patients who decline participation will be considered an indicator of recruitment feasibility of the intervention. The number of withdrawals and their reasons will be reported separately from initial refusals and losses occurring during the intervention. A CONSORT-style participant flow diagram adapted to the non-randomized study design will be provided to summarize participant flow.

Secondary objectives focus on evaluating preliminary efficacy across clinical, functional, and psychological domains. Secondary outcomes encompass changes in global functioning as measured by the Global Assessment of Functioning (GAF) scale, mental health literacy regarding the disorder and its pharmacological treatment assessed via the Mental Health Knowledge Schedule (MAKS), problem-solving skills, emotion regulation capacity evaluated using the Difficulties in Emotion Regulation Scale (DERS), and coping strategies measured by the Coping Orientation to Problems Experienced (COPE-NVI). Additional clinical secondary outcomes include anxiety and depression symptoms assessed with the Hospital Anxiety and Depression Scale (HADS), severity of psychopathology measured by the Brief Psychiatric Rating Scale (BPRS), overall illness severity and improvement evaluated through the Clinical Global Impressions scales (CGI-S and CGI-I), internalized stigma assessed using the Internalized Stigma of Mental Illness scale (ISMI), and cognitive functioning evaluated with the Montreal Cognitive Assessment (MoCA).

Efficacy analyses will follow a modified intention-to-treat (mITT) approach, including all enrolled prospective participants and eligible historical control participants according to the group to which they were assigned. A per-protocol analysis, restricted to prospective participants who complete the serious games intervention according to protocol and historical controls with complete follow-up data, will be conducted as a sensitivity analysis.

Secondary continuous outcomes will be analyzed using linear mixed-effects models (LMMs) tailored to the distribution of each outcome variable. Models will include fixed effects for treatment group, time, and the group x time interaction, which represents the primary effect of interest. A random intercept for participant will be included to account for within-subject correlation across repeated measurements, with additional random slopes for time explored based on model fit. To control for potential baseline confounding inherent to the non-randomized study design with a historical control group, relevant baseline covariates, including age, sex, baseline outcome scores, and diagnosis, illness severity and duration, previous hospitalization will be included as adjustment variables in multivariable models. Variables that will be thought of changing across time, and therefore possibly changing the results, will be also used as covariables in the regression models.

Missing outcome data will be handled primarily through the linear mixed-effects modelling framework, which uses all available observations under the missing-at-random (MAR) assumption. If the proportion of missing data exceeds 15%, multiple imputation by chained equations (MICE) will be performed as a sensitivity analysis. Twenty imputed datasets will be generated. The imputation model will include treatment group, assessment time, all study outcomes, age, sex, diagnosis, baseline outcome values, and other relevant sociodemographic and clinical variables that may be associated with the probability of missingness. The longitudinal structure of the data and treatment group will be retained in the imputation model. The adequacy of the imputation procedure will be assessed by examining convergence diagnostics, including trace plots across imputation iterations, and by comparing the distributions of observed and imputed values to identify potentially implausible imputations. Results from analyses based on the imputed datasets will be compared with those obtained from the primary mixed-effects models using available data and from complete-case/per-protocol analyses as sensitivity analyses. Consistency of the direction and magnitude of effect estimates and their 95% confidence intervals across these approaches will be used to assess the robustness of the findings.

All statistical analyses will be performed using STATA Software, version 19. The level of statistical significance will be set at *p* = 0.05, two-tailed, for all comparisons.

## Results

We expect the implementation of serious games will be feasible and overall, well accepted by participants. We expect that the integration of serious games could improve participants' engagement, strengthen outcomes reached with standard psychoeducation, by improving the mental health literacy and skills (e.g., emotion regulation, more adaptive and flexible coping strategies) acquired during the standard psychoeducational intervention. By providing an engaging and interactive way to complete homework assignments, the serious games are expected to enhance participants' adherence to between-session activities, promote greater practice of the skills introduced during the sessions, and facilitate their consolidation and transfer to everyday life. As secondary effects, we hypothesize that participants receiving the serious game intervention will show greater improvements especially in global functioning and severity of psychopathological symptoms.

## Discussion

Digital mental health interventions (DMHIs) are increasingly recognized as valuable adjuncts to conventional psychiatric care and are already changing mental health care in exciting ways, each accompanied by an early but promising evidence base particularly for young people and individuals with SSDs, who often experience barriers to treatment engagement and continuity of care (Torous et al., 2021). Recent evidence suggests that DMHIs can be valuable tools for improving accessibility, supporting self-management, and facilitating personalized care pathways, although their effects on core psychotic symptoms remain modest and largely depend on sustained user engagement (Liverpool et al., 2025).

Within this rapidly evolving field, serious games have attracted growing interest because they particularly address one of the main limitations of conventional psychosocial interventions, namely declining adherence over time. Unlike conventional psychoeducational approaches based primarily on passive information delivery, psychoeducational serious games combine therapeutic content with interactive learning, immediate feedback, and experiential practice (Gómez-Cambronero et al., 2024). Rather than merely delivering information, serious games encourage users to apply acquired knowledge through simulated situations, problem-solving tasks, and decision-making processes, thereby supporting experiential learning and behavioral rehearsal. Serious games are games specifically designed to achieve educational objectives beyond entertainment, whereas gamification refers to the incorporation of game design elements—such as points, levels, badges, avatars, challenges, and rewards—into non-game contexts (Deterding et al., 2011). This distinction is particularly relevant because the mechanisms through which these approaches influence behavior are likely to differ. Serious games rely on immersive narratives, experiential learning, and repeated behavioral practice, whereas gamification primarily seeks to enhance motivation within existing interventions through structured reinforcement and feedback.

The potential effectiveness of serious games may be explained through established psychological theories. Self-Determination Theory proposes that sustained behavioral engagement depends on satisfying three fundamental psychological needs: autonomy, competence, and relatedness (Ryan and Deci, 2000). Serious games may simultaneously address all three dimensions by allowing users to make meaningful choices, progressively master increasingly challenging tasks, and interact with virtual or real social environments. Similarly, Flow Theory suggests that optimal engagement occurs when perceived challenges closely match an individual's skills, generating deep concentration, intrinsic enjoyment, and persistence (Salanova et al., 2014). These theoretical frameworks offer a more sophisticated explanation for the success of game-based interventions than simple behavioral reward models based exclusively on points or badges.

Recent reviews suggest that serious games can enhance motivation, participation, and mental health literacy, particularly among adolescents and young adults who are highly familiar with digital environments (Zeiler et al., 2025). However, the evidence on the efficacy and effectiveness of serious game interventions in this field should be regarded as preliminary. More rigorously planned studies, including RCTs and real-world trials, are needed.

The broader youth mental health literature further supports this perspective. A recent review of reviews synthesizing 218 digital game interventions concluded that serious games generally produce positive effects on emotional wellbeing, resilience, mental health literacy, and engagement, although the quality of evidence remains heterogeneous and relatively few studies have isolated the contribution of individual gamification components (Vié et al., 2024).

The evidence supporting game-based interventions in schizophrenia is also expanding. The most recent scoping review, including 82 studies published between 2014 and 2025 (Yang et al., 2025), found that serious games, gamified applications, and commercial games were associated with improvements in social cognition, cognitive performance, negative symptoms, and user engagement. Notably, serious games demonstrated the strongest effects on social cognition, whereas gamified mobile applications appeared particularly effective in improving motivation and treatment adherence. Nevertheless, the review also highlighted important methodological limitations, including small sample sizes, short follow-up periods, and the limited use of theory-driven intervention design, and emphasized that these technologies should currently be considered complementary rather than stand-alone therapeutic approaches.

Moreover, an important gap persists because most digital game interventions have been developed either for common mental disorders, such as anxiety and depression, or for adults with chronic schizophrenia. Consequently, relatively few interventions specifically target adolescents and young adults with first-episode psychosis or SSD, despite this period representing a critical window for early intervention. Given that early engagement substantially influences long-term clinical and functional outcomes, developing digital psychoeducational interventions that combine evidence-based therapeutic principles with engaging game mechanics represents a promising direction for future research (Dewhirst et al., 2022; Ferrari et al., 2022; Yang et al., 2025).

Psychoeducation remains one of the most promising evidence-based psychosocial interventions for schizophrenia, consistently improving illness knowledge, relapse prevention, treatment adherence, and family outcomes (Lincoln et al., 2007; Xia et al., 2011). However, conventional psychoeducation frequently relies on didactic formats that may be insufficiently engaging for younger individuals experiencing first-episode psychosis or early schizophrenia. Digital psychoeducational interventions incorporating serious games may overcome these limitations by transforming passive learning into interactive and personalized experiences that encourage active participation, repeated practice, and autonomous exploration of illness-related content. In this respect, game-based psychoeducation may better align with contemporary recovery-oriented models, which emphasize empowerment, self-efficacy, and collaborative care rather than simple information transfer.

Overall, the available literature suggests that serious games have the potential to extend the benefits of digital mental health beyond simple symptom monitoring by fostering motivation, experiential learning, and sustained engagement. However, future studies should move beyond feasibility research toward adequately powered randomized controlled trials with longer follow-up and standardized outcome measures. Greater attention should also be devoted to participatory co-design involving young people with lived experience, ensuring that digital interventions are not only technologically innovative but also clinically meaningful, developmentally appropriate, and recovery oriented.

The present protocol extends the existing literature on serious games mental health interventions for SSD by proposing an approach that combines psychoeducational contents with serious gamification strategies within a digital format. While previous studies have demonstrated the feasibility and potential benefits of digital interventions, including serious games and gamified approaches, these interventions have often focused on specific outcomes, such as cognitive remediation, symptom management, or treatment adherence, and few of those interventions specifically targeted adolescents and young adults with first-episode psychosis or schizophrenia spectrum disorders. Our protocol aims to provide further evidence on serious game interventions tailored to young inpatients with schizophrenia spectrum disorders by providing an analysis of several outcomes for its evaluation. By explicitly situating the intervention within the current evidence base, this study seeks to contribute to the development of digital interventions for young people with SSD and CHR-P while addressing some limitations identified in previous research, such as the need for more standardized and robust evaluation.

## Strengths and limitations of this study

The current research protocol aims at integrating an interactive and game-based digital approach within the context of youth mental health inpatient settings. It examines a novel and engaging youth-friendly approach which potentially can increase participants' attractiveness and treatment retention, particularly in acute settings. Therefore, its major strength is represented by the creation of an integrated digital approach specifically designed to adolescents and young adults, more prone to use digital tools compared to standard treatments. Moreover, this could imply that it has the potential to engage inpatients at the very early phases of mental illness, including CHR-P. Another strength is represented by the fact that the study has been designed to be run within an acute and transitional psychiatric hospitalization. By doing so, we wanted to test it under the least favourable conditions, such as interfering variables like duration of hospitalization, symptoms severity and patient's compliance which may interfere with the acceptability and connected to it, the completion of the intervention.

The primary goal of the present research is to demonstrate that this approach is feasible independently by clinical variability in terms of psychopathological severity of SSD and/or CHR-P subjects and setting unfavorable conditions.

However, there are study limitations which should also be discussed. Firstly, the study is only based on a single-center sample. Being a feasibility and preliminary effectiveness study, it is expected to replicate the study using a multicentre study design whereas positive outcomes will be reached. Secondly, the study involves a relatively small sample of inpatients. Therefore, the intention is to furtherly increase the sample size by using a study design that allows for stratifying for different diagnostic subgroups, for example including severe mood disorders. Another limitation concerns the absence of medium or long-term follow-up. A longitudinal design should be considered in the future. Other (prospective) limitations include: (a) the limited causal inference due to the absence of randomization. The findings related to the secondary outcomes will consequently be interpreted as preliminary and hypothesis-generating, providing a basis for a future adequately powered randomized controlled trial; (b) the use of a historical control group which may introduce temporal confounding e uncertainty about distinguishing intervention effects from potential secular changes. It should be noted however that for the present study, to minimize this potential source of bias, the historical control cohort was selected from patients treated during the three months immediately preceding the introduction of the serious games, thus limiting the time interval between the two cohorts. However, we acknowledge that these comparisons cannot completely exclude unmeasured potential changes in clinical practice over time. Because we have not systematically quantified all potential secular changes in service organization or clinical practice, between-group differences observed will be explained cautiously, without attributing them exclusively to the serious games' intervention; at the same time, the use of a historical control group can imply other bias related to seasonal effects or hospital policies changes. For the present preliminary study, these prospective confounders were not systematically collected as study variables. It must be said, however, that, based on the information reported by the Unit's staff throughout the study period, the rehabilitation setting, the standard treatment protocol, healthcare professionals, and clinical pathways were planned to remain unchanged; (c) the short observation period. However, the timing of the outcome assessments was defined according to the clinical setting of the Unit of Clinical Psychiatry involved in the present study and was broadly comparable to the approach described by the staff of that Unit in their recent study where the psychoeducational intervention was delivered during hospitalization and assessment was scheduled in relation to the completion of the intervention. Accordingly, in our study, baseline assessment is performed before the intervention, whereas the post-intervention assessment is performed at the end of the intervention, generally coinciding with hospital discharge. When this won't be feasible, the assessment is planned to be completed at the first scheduled post-discharge follow-up visit, usually approximately 15 days after discharge; (d) the limited external validity of the results, as the study is conducted in a specific clinical setting and involves a selected sample of patients meeting predefined eligibility criteria. Therefore, the findings may not be directly generalizable to other psychiatric settings or to patients with different clinical or sociodemographic characteristics. Moreover, the exclusion of patients with moderate-to-severe cognitive deficits or significant learning difficulties may further restrict the generalizability of the findings to patients with greater cognitive or functional impairment; (e) the participants' digital literacy and familiarity with digital technologies which may influence the use of serious games. Although the intervention was designed to be accessible and easy to use, differences in previous experience with digital devices could affect participants' engagement with and perceived usability of the games; (f) the large number of psychological measures. However, the selection of those measures was based on the routine assessment procedure already established in the psychiatric Unit involved in the study. Those measures are routinely administered in the Unit as part of the standard assessment of psychiatric inpatients. Their use in the present study allows the assessment to remain consistent with the clinical practice already adopted in the Unit. Regarding respondent burden and feasibility, the fact that these instruments have been routinely administered for several years to psychiatric inpatients in that Unit provides practical evidence that the assessment procedure is feasible even for patients in the acute phase; (g) the absence of a study-specific safety monitoring the serious-game intervention. In particular, the study does not include a dedicated adverse-event reporting system, predefined criteria for clinical deterioration, specific discontinuation criteria, or a separate psychiatric emergency protocol related to the intervention. Participants are psychiatric inpatients and therefore remain under the routine clinical supervision of the treating team throughout their hospitalization. Any clinical deterioration or psychiatric emergency would be managed according to the usual clinical care procedures of the psychiatric unit; (h) as a preliminary study, a formal procedure of intervention fidelity assessment was not provided. A more structured procedure of intervention fidelity assessment including information about staff training, fidelity checklist, supervision procedures, protocol adherence and protocol deviations will be incorporated in a larger future trial.

An additional limitation of the present study is the absence of a qualitative evaluation of participants' and healthcare professionals' experiences. Qualitative methods, such as semi-structured interviews or open-ended questionnaires, could provide valuable information regarding usability, perceived benefits, barriers to engagement, and suggestions for improving the serious games. Future studies should adopt a mixed-methods approach to complement quantitative findings and support the refinement of the intervention before larger-scale clinical evaluation. Moreover, future studies should also integrate pharmacological variables in the statistical analyses, as potential interfering/controlling factors to be managed, to establish whether psychopathology, functioning, cognition improvement, as well as treatment engagement and attrition rate are influenced by the type of pharmacological treatment adopted. Finally, one important consideration is the potential influence of the novelty effect. The potential higher satisfaction which may be reported by participants using the serious game may be partly determined by the appeal of a new and interactive digital format rather than by the psychoeducational content itself. As the present study does not include measures specifically designed to assess or control novelty or technology acceptance, it is not possible to determine the extent to which this factor may contribute to the observed differences. Future research should include active digital comparison conditions, measures of user expectations and engagement, and longer follow-up periods to evaluate whether the observed benefits persist once the initial novelty of the intervention has diminished.
