# Supplementary material for: Digital psychoeducational rehabilitation supported by serious games for psychiatric inpatients: a protocol study
Source: Front Psychol. 2026 Sep 18;17:1881403. doi: 10.3389/fpsyg.2026.1881403 (PMC13630922; doi:10.3389/fpsyg.2026.1881403)
Supplement: Supplementary file 1 [file Data_Sheet_1.PDF]

Table 1. Topics of the psychoeducational intervention sessions

- **Basic Emotions**: The objectives will be to provide information on Ekman's five primary emotions, including contextualizing them with facial expressions and teaching their recognition, as well as teaching the ABC model (Ellis 1957; Beck 1975) for recognizing unpleasant emotions and corresponding thoughts. A relevant serious game will be presented and encouraged to be played as homework, to consolidate the information received and the knowledge acquired on the topic.
- **Anxiety and Sadness**: The objectives will be to provide information on how to distinguish and recognize normal and pathological anxiety and sadness, identify which situations trigger these emotions, and teach behaviors that reduce susceptibility to anxiety. The relevant serious game will be presented and encouraged to be played, which includes informational material that reiterates the themes discussed during the meeting to consolidate the information received and the knowledge acquired on the topic.
- **Unusual Experiences**: The meeting will feature an initial fictitious clinical case to introduce the topic. Subsequently, information will be provided regarding the various types of "unusual experiences" that patients typically experience before or during hospitalization, along with possible coping strategies. The relevant serious game will be proposed and encouraged to be played to consolidate the information received and the knowledge acquired on the topic.
- **Addictions:** The objectives of this module are to provide information on substances to improve awareness and inform patients of the risks associated with their use. It also provides information on alternative strategies for substance use and possible behavioral addictions (including digital addiction). The relevant serious game will be proposed and encouraged to be played, allowing the information received and the knowledge acquired on the topic to be consolidated.
- **Sleep:** The objectives will be to provide information on sleep, proper hygiene, factors that influence it (positively and negatively), and strategies useful for improving it. The relevant serious game will be proposed and encouraged to be played, which will likely include the exercise of completing a personal sleep diary.
- **Treatment:** During this session, patients will be informed about available and unavailable pharmacological therapies, discussing the importance of taking their medications regularly and identifying the best strategies in the event of side effects. Playing the relevant serious game will be proposed and encouraged to consolidate the information received and the knowledge acquired on the topic.
- **Communication skills:** The objectives will be to encourage learning effective communication skills (expressing pleasant and unpleasant emotions, making positive requests, and active listening). A relevant serious game will be proposed and encouraged, which, for example, could involve asking the patient to identify/recognize critical situations in which the strategies explained by the professionals could be used.
- **Problem Solving:** The goal of this session is to learn the components of problem solving. This is a structured process for addressing complex situations, primarily consisting of: problem finding, clear definition (problem setting), root cause analysis (problem analysis), solution generation, decision making, and finally implementation and monitoring of the solution (active problem solving). Again, a serious game will be used to propose a problem to be solved by practicing all the components of problem solving.
- **Social skills:** The goal is to develop certain social skills necessary in interpersonal situations to communicate with others appropriately and effectively. Playing a relevant serious game will be proposed and encouraged, allowing students to consolidate the information received and the knowledge acquired on the topic.
